# Supplementary material for: Impaired cardiac performance, protein synthesis, and mitochondrial function in tumor-bearing mice
Source: PLoS One. 2019 Dec 18;14(12):e0226440. doi: 10.1371/journal.pone.0226440 (PMC6919625; doi:10.1371/journal.pone.0226440)
Supplement: S1 File — (DOCX) [file pone.0226440.s001.docx]

**S1 Supporting Information File**

**Supplemental Figure A.** Photomicrographs of sections of ventricular tissue obtained from mice injected with PBS vehicle (panels A-C) or LLC1 cells (panels D-F), and stained with Masson trichrome stains. Note the absence of fibrosis in ventricular sections from LLC1 injected mice. Scale bar = 100 μm.

**
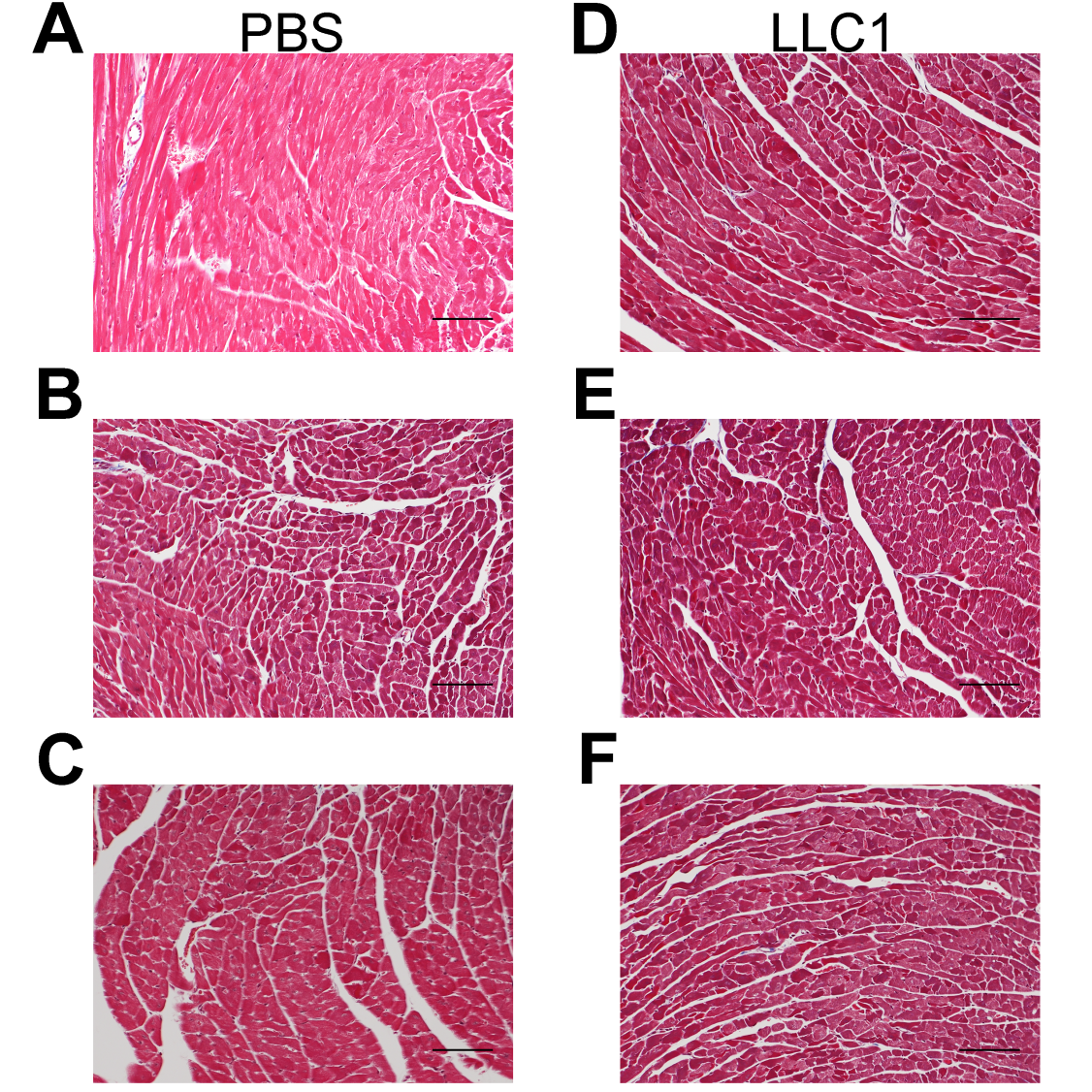
**

**Supplemental Table A.** mRNAs encoding mitochondrial respiratory chain complexes in the hearts of 14-day LLC1 tumor-bearing male mice.

| **Transcripts of mitochondrial respiratory chain complexes altered in tumor-bearing mice** | | |
| --- | --- | --- |
| **Gene Name** | **Fold Change** | **FDR** |
| Ndufs7, NADH: ubiquinone oxidoreductase core subunit S7 | 1.338 | 0.002282 |
| Ndufa7, NADH: ubiquinone oxidoreductase subunit A7 | 1.322 | 0.001476 |
| Cox6a2, cytochrome c oxidase subunit 6A2 | 1.317 | 0.004653 |
| Uqcr11, ubiquinol-cytochrome c reductase, complex III subunit XI | 1.282 | 0.004615 |
| Ndufa13, NADH: ubiquinone oxidoreductase subunit A13 | 1.261 | 0.016382 |
| Ndufv1, NADH: ubiquinone oxidoreductase core subunit V1 | 1.259 | 0.006473 |
| Atp5d, ATP synthase, H+ transporting, mitochondrial F1 complex, delta subunit | 1.252 | 0.015208 |
| Ndufa1, NADH: ubiquinone oxidoreductase subunit A1 | 1.233 | 0.019105 |
| Ndufa6, NADH: ubiquinone oxidoreductase subunit A6 | 1.228 | 0.028311 |
| Ndufa3, NADH: ubiquinone oxidoreductase subunit A3 | 1.224 | 0.038189 |
| Cox8b, cytochrome c oxidase subunit 8B | 1.223 | 0.039008 |
| Ndufb8, NADH: ubiquinone oxidoreductase subunit B8 | 1.221 | 0.011597 |
| Ndufa11, NADH: ubiquinone oxidoreductase subunit A11 | 1.215 | 0.040269 |
| Uqcr10, ubiqunol-cytochrome c reductase, complex III subunit X | 1.208 | 0.045979 |
| Sdhb, succinate dehydrogenase complex iron sulfur subunit B | 1.196 | 0.044406 |

**Supplemental Table B.** Significantly altered mRNAs encoding autophagy-related genes in the hearts of 14-day LLC1 tumor-bearing male mice.

| **Autophagy-related transcripts altered in tumor-bearing mice** | | |
| --- | --- | --- |
| **Gene Name** | **Fold Change** | **FDR** |
| Gabarapl1, gamma-aminobutyric acid receptor-associated protein-like 1 | 1.485 | 1.98E-08 |
| Sqstm1, sequestosome-1 | 1.287 | 0.009269 |
| Ulk1, unc-51 like kinase 1 | 1.279 | 0.020312 |
| Bnip3, BCL2/adenovirus E1B interacting protein 3 | 1.270 | 0.0058 |
| Atg13, autophagy-related protein 13 | 1.241 | 0.001916 |
| Map1lc3a, microtubule-associated protein 1 light chain 3 alpha | 1.240 | 0.016641 |
| Map1lc3b, microtubule-associated protein 1 light chain 3 beta | 1.223 | 0.005151 |
| Atg14, autophagy-related protein 14 | 1.211 | 0.047241 |
| Gabarap, gamma-aminobutyric acid receptor-associated protein | 1.195 | 0.016318 |

**Supplemental Table C.** mRNAs encoding proteolysis-related genes altered in the hearts of 14-day LLC1 tumor-bearing male mice.

| **Proteolysis-related mRNA transcripts altered in tumor-bearing mice** | | |
| --- | --- | --- |
| **Gene Name** | **Fold Change** | **FDR** |
| Psme2b, proteasome activator complex subunit 2 | 0.558 | 0.018842 |
| Rnf144a, E3 ubiquitin-protein ligase RNF144A | 0.648 | 5.71E-05 |
| Mmp2, 72 kDa type IV collagenase | 0.654 | 1.56E-05 |
| Ctss, cathepsin S | 0.691 | 0.026646 |
| Fbxl16, F-box/leucine rich-repeat protein 16 | 0.701 | 0.038901 |
| Rnf138, E3 ubiquitin-protein ligase RNF138 | 0.760 | 0.030079 |
| Senp1, sentrin-specific protease 1 | 0.806 | 0.039524 |
| Smurf2, E3 ubiquitin-protein ligase SMURF2 | 0.842 | 0.038502 |
| Psma7, proteasome subunit alpha type-7 | 1.171 | 0.047241 |
| Ctsf, cathepsin F | 1.186 | 0.010763 |
| Aurkaip1, aurora kinase A-interacting protein | 1.211 | 0.034992 |
| Psmc1, 26S proteasome regulatory subunit 4 | 1.212 | 0.003606 |
| Spg7, paraplegin | 1.215 | 0.010203 |
| Psmb10, proteasome subunit beta 10 | 1.239 | 0.003173 |
| MuRF1, muscle RING-finger protein-1 | 1.265 | 0.004397 |
| Rnf144b, E3 ubiquitin-protein ligase RNF144B | 1.277 | 0.016413 |
| Psmd4, 26S proteasome non-ATPase regulatory subunit 4 | 1.288 | 0.000815 |
| Rnf114, E3 ubiquitin-protein ligase RNF114 | 1.303 | 7.09E-05 |
| Fbxo32, F-box only protein 32 | 1.364 | 0.012257 |
| Rnf166, RING finger protein 166 | 1.380 | 2.68E-05 |
| Fbxo31, F-box only protein 31 | 1.412 | 8.48E-05 |

**Supplemental Table D.** mRNAs encoding apoptosis-related genes altered in the hearts of 14-day LLC1 tumor-bearing male mice.

| **Apoptosis-related mRNA transcripts altered in tumor-bearing mice** | | |
| --- | --- | --- |
| **Gene Name** | **Fold Change** | **FDR** |
| Pik3cg, phosphatidylinositol 4,5-bisphosphate 3-kinase catalytic subunit gamma isoform | 0.566 | 0.00192 |
| Tnfsf10, tumor necrosis factor ligand superfamily member 10 | 0.695 | 0.008275 |
| Casp8, caspase 8 | 0.703 | 0.027029 |
| Birc3, baculoviral IAP repeat containing 3 | 0.733 | 0.01319 |
| Pik3r3, phosphatidylinositol 3-kinase regulatory subunit gamma | 0.737 | 0.000551 |
| Apaf1, apoptotic protease-activating factor 1 | 0.744 | 0.006145 |
| Akt3, RAC-gamma serine/threonine-protein kinase | 0.778 | 0.001882 |
| Casp7, caspase 7 | 0.782 | 0.010435 |
| Endod1, endonuclease domain-containing 1 protein | 0.788 | 0.041991 |
| Xiap, X-Linked inhibitor of apoptosis | 0.829 | 0.010693 |
| Fas, fas cell surface death receptor | 1.236 | 0.049854 |
| Pik3r1, phosphatidylinositol 3-kinase regulatory subunit alpha | 1.245 | 0.020027 |
| Bcl7b, B-cell CLL/lymphoma 7 protein family member B | 1.257 | 0.011094 |
| Nfkbia, NF-kappa-B inhibitor alpha | 1.292 | 0.02006 |
| Pik3r2, Phosphatidylinositol 3-kinase regulator subunit beta | 1.306 | 0.020866 |

**Supplemental Table E.** Cardiac ventricular mRNAs changed in tumor-bearing mice vs. vehicle injected mice with a FDR = false discovery rate <0.05 and with fold change <0.5 or > 1.5.

| **Cardiac mRNA transcripts decreased in tumor-bearing mice** | | |
| --- | --- | --- |
| **Gene Name** | **Fold Change** | **FDR** |
| Top2a, DNA topisomerase. | 0.098134 | 1.30E-09 |
| Mki67,Proliferation marker protein Ki-67 | 0.127234 | 1.02E-07 |
| Anln, Anillin. | 0.186818 | 7.73E-12 |
| Prnd, Prion-like protein doppel | 0.217861 | 6.12E-18 |
| Prc1,Protein regulator of cytokinesis 1. | 0.261762 | 5.47E-06 |
| Mycn,N-myc proto-oncogne. | 0.285281 | 7.42E-10 |
| Thbs1, Thrombospondin-1. | 0.295543 | 0.001755 |
| Cxcl9, C-X-C motif chemokine 9. | 0.307017 | 1.28E-13 |
| Akap5, A-kinase anchor protein 5. | 0.308684 | 4.88E-16 |
| Aplnr, Apelin receptor. | 0.310931 | 3.68E-26 |
| Tfrc, Transferrin receptor. | 0.311658 | 0.000106 |
| Kit, Mast/stem cell growth factor receptor Kit | 0.315407 | 9.69E-07 |
| Prdm1, PR domain zinc finger protein 1. | 0.328736 | 3.62E-14 |
| Egr1, Early growth response protein 1. | 0.341496 | 0.000386 |
| Cd109, CD109 antigen. | 0.358862 | 5.84E-09 |
| Mest, Mesoderm-specific transcript protein. | 0.358917 | 2.23E-07 |
| Bcl6b, B-cell CLL/lymphoma 6 member B protein. | 0.374137 | 2.25E-18 |
| Arhgap11a, Rho GTPase-activating protein 11A. | 0.381553 | 1.98E-08 |
| Gas2l3, GAS2-like protein 3. | 0.389585 | 4.92E-06 |
| Postn, Periostin. | 0.391896 | 6.53E-10 |
| Mfap4,Microfibril-associated glycoprotein 4. | 0.403241 | 0.014268 |
| Meox1, Homeobox protein MOX-1. | 0.403697 | 4.55E-17 |
| Cd84,SLAM family member 5. | 0.413139 | 9.30E-07 |
| Ift122, Intraflagellar transport protein 122 homolog. | 0.414754 | 3.47E-07 |
| Apln, Apelin. | 0.418025 | 4.82E-14 |
| Scml4, Sex comb on midleg-like protein 4. | 0.421431 | 7.89E-05 |
| Adamts12, A disintegrin and metalloproteinase  with thrombospondin motifs 12 | 0.424766 | 2.14E-09 |
| Uhrf1, E3 ubiquitin-protein ligase UHRF1. | 0.429056 | 0.001541 |
| Cilp, Cartilage intermediate layer protein 1. | 0.429099 | 0.008309 |
| Spon2,Spondin-2. | 0.432294 | 0.006566 |
| Neurl3, Neurl3. E3 ubiquitin-protein ligase. | 0.43349 | 7.78E-11 |
| Nes, Nestin. | 0.436866 | 3.91E-16 |
| Nrep, Neuronal regeneration-related protein. | 0.440679 | 8.33E-17 |
| Ccr5, C-C chemokine receptor type 5. | 0.449128 | 0.031396 |
| Ttyh2, Protein tweety homolog 2. | 0.450238 | 4.38E-08 |
| Arsi, Arysulfatase I. | 0.458228 | 3.17E-05 |
| Rrm2, Ribonucleoside-diphosphate reductase subunit M2. | 0.466214 | 2.10E-05 |
| Sez6l2, Seizure 6-like protein 2. | 0.468229 | 4.44E-05 |
| Sox9, Transcription factor SOX-9. | 0.468711 | 0.000936 |
| Ces2e, Pyrethroid hydrolase Ces2e. | 0.470191 | 4.65E-05 |
| Papln, Papilin | 0.472058 | 2.30E-06 |
| Cx3cr1, CX3C chemokine receptor 1. | 0.473648 | 0.010322 |
| Vcan, Versican core protein. | 0.475213 | 3.09E-12 |
| Ccnd1, G1/S-specific cyclin-D1. | 0.47673 | 7.68E-13 |
| Cd40, CD40 ligand. | 0.478537 | 8.32E-07 |
| Col14a1, Collagen alpha-1(XIV) chain. | 0.4834 | 0.000174 |
| Marcksl1, MARCKS-related protein. | 0.483565 | 6.13E-06 |
| Fscn1, Fascin. | 0.488509 | 7.07E-08 |
| Zc3hav1l, Zinc finger CCCH-type antiviral protein 1-like. | 0.488565 | 2.68E-05 |
| Kctd12b, Potassium channel tetramerisation domain | 0.489102 | 1.95E-19 |
| containing 12b, isoform CRA_a. |  |  |
| Loxl2, Lysyl oxidase homolog 2. | 0.489906 | 1.61E-18 |
| Tnfaip8l1, Tumor necrosis factor α-induced | 0.491861 | 3.39E-05 |
| protein 8-like protein 1. |  |  |
| Vash1, Tubulinyl-Tyr carboxypeptidase 1. | 0.498803 | 2.96E-10 |
| Map1b, Microtubule-associated protein 1B. | 0.499141 | 1.95E-08 |
| **Transcripts increased in tumor-bearing mice** | | |
| Gadd45b, Growth arrest and DNA damage-inducible protein | 2.041177 | 1.01E-06 |
| GADD45 beta |  |  |
| Inmt, Indolethylamine N-methyltransferase. | 2.174738 | 0.001204 |
| C4b, Complement C4-B | 2.227294 | 1.42E-07 |
