# Supplementary figures and images for: Impaired cardiac performance, protein synthesis, and mitochondrial function in tumor-bearing mice
Source: PLoS One. 2019 Dec 18;14(12):e0226440. doi: 10.1371/journal.pone.0226440 (PMC6919625; doi:10.1371/journal.pone.0226440)

**A**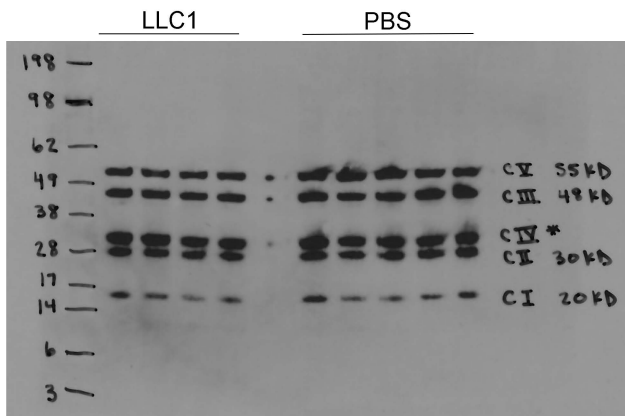**B**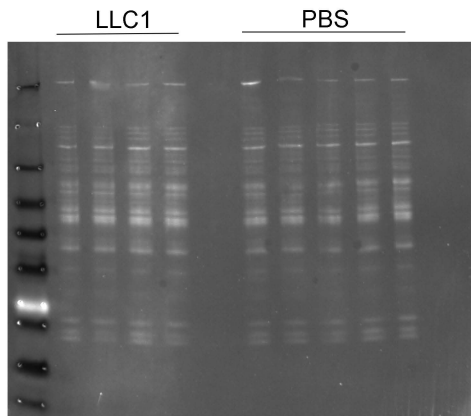

Supplement: S1 Raw Images — (PDF) [file pone.0226440.s002.pdf]
