# Supplementary material for: Impaired cardiac performance, protein synthesis, and mitochondrial function in tumor-bearing mice
Source: PLoS One. 2019 Dec 18;14(12):e0226440. doi: 10.1371/journal.pone.0226440 (PMC6919625; doi:10.1371/journal.pone.0226440)
Supplement: Attachment — Submitted filename: Response to reviewers.docx [file pone.0226440.s003.docx]

**Response to reviewers.**

**ALL CHANGES HAVE BEEN UNDERLINED IN THE REVISED MANUSCRIPT.**

**Reviewer #1:**

***Summary of findings:*** In this article the authors investigated the underlying mechanisms of cancer-induced cardiac dysfunction using a tumor-bearing mouse model. They observed that male tumor-bearing mice manifest the reduced ejection fraction and ventricular wall thickness. However, these phenomena do not occur in female tumor-bearing mice. They further hypothesize that reduced ventricular wall thickness and ejection fraction observed in male tumor-bearing mice could be associated with impaired protein synthesis and mitochondria function. Evidence showed that protein synthesis is reduced and mitochondria morphology and function are changed as well in male tumor-bearing mice. The authors also performed RNA-seq and found differentially changed genes are associated with protein synthesis as well as mitochondria function. Therefore, they concluded that cardiac atrophy in male tumor-bearing mice is associated with reduced protein synthesis and impaired contractile function is related to altered mitochondria function. Several comments are listed below:

**Comments and responses:**

**Comment 1.** There is no detailed discussion why authors observed cancer-induced cardiac dysfunction in male, but not in female.

*Response:* We discuss why female mice do not respond in a manner similar to male mice. Previous work (Cosper PF, Leinwand LA. Cancer causes cardiac atrophy and autophagy in a sexually dimorphic manner. Cancer Res. 2011;71(5):1710-20) has shown that this is mediated by the estrogen receptor. The use of an estrogen receptor blocking agent, fulvestrant, in female mice with cancer, is associated with a response that is similar to that observed in male mice. We have now added text discussing this issue.

**Comment 2.** By looking at the RNA-seq data, actually, almost all genes listed in the tables are not significantly changed (<1.5), which is not convincing that these subtle changes are associated with cardiac dysfunction. I will suggest authors also test the protein levels with immunoblotting using hall mark genes associated with protein synthesis and mitochondria function to validate the RNA-seq data.

*Response:* We now provide immunoblot data showing that changes in the mRNAs for components of the mitochondrial respiratory chain complex are paralleled by changes observed in protein components of the respiratory chain complex assessed by (new figure 6).

**Comment 3.** If possible, pathological data such as fibrosis could be added which would be more supportive for the conclusion

*Response:* We examined cardiac tissues by histology and did not notice an increase in fibrosis. A statement to this effect is now included in the manuscript.

**Comment 4.** The resolution of the figures is not high.

*Response:* This has now been corrected.

**Comment 5.** Figure 1-4 can be integrated into two figures. Characterization of male mice can be consolidated into figure 1, and female data in figure 2

*Response:* This has now been done.

**Comment 6.** In figure 5, authors used over 9 animals in B and C, but why only 4 animals were used in A?
*Response:* We had only 4 samples available to us for analysis of ^13^C phenylalanine incorporation into protein.

**Comment 7.** I would suggest moving the tables with next generation sequencing data to supplement.

*Response:* We have moved the next generation sequencing data into the supplement.

**Reviewer #2**

***Summary of findings:*** In the present manuscript, Kumar and colleagues aimed to address pathological changes in the heart under tumor-bearing conditions. The authors utilized lung tumor as an example to induce cachexia and evaluated heart function. They showed, in a convincing way, cardiomyopathy emerged at 14 days post tumor inoculation. Moreover, the heart growth was impaired. The authors went further to show mitochondria had certain abnormalities and maximal oxygen consumption was reduced.

**Comment 1.** The studies were thoughtfully designed and nicely executed. However, the conclusions suffer from questions in novelty. Tumor-related cachexia and related heart disease has been noted for years. Moreover, previous studies have suggested the increase in atrophy and decrease in mitochondrial action may contribute. For a few examples, please see, Spring H, 2014, EHJ; Tian M, 2010, International J of Oncology, etc. Therefore, the present study may not provide significant conceptual advances in the field of cachexia-associated heart disorder.

*Response:* Previous studies have demonstrated heart failure in various animal models injected with different types of tumor cells. Our study, however, significantly adds to knowledge about mechanisms responsible for reduced cardiac function. First, to our knowledge, we are unaware of studies performed previously with LLC1 cells, a model of lung non-small cell tumors. Second, we are unaware of studies in which investigators have examined the effect of tumor conditioned medium on mitochondrial oxygen uptake and the effect of tumor cell implantation on cardiac protein synthesis. Third, using whole transcriptome sequencing, we show reductions in the expression of mRNAs which encode proteins that affect cardiac function and growth e.g. *Apelin*, *Apelin receptor*, *Sox 9,* and *Egr1*. Increases in the expression of mRNAs involved in the regulation of growth arrest and apoptosis such as *Gadd45B* is noted in tumor-bearing mice.

**Comment 2.** In the last figure, conditioned media (CM) from tumor cells inhibited OCR. Is this due to inflammatory cytokines? If so, how to narrow down the candidates?

*Response:* We thank the reviewer for the comment. We do not know the mediator for a reduction in mitochondrial oxygen consumption rate. This is an ongoing project in our laboratory at the present time. We have added text to address this issue and potential paths that can be taken to isolate such a mediator.

**Comment 3.** The authors have done thorough analysis for the RNA-Seq data. What about changes at the protein level? Such as atrophy-related proteins and the growth signaling pathway (i.e. mTOR).

*Response:* Please see the response to comment 2 by reviewer 1. We now provide data showing that changes in the mRNAs for components of the mitochondrial respiratory chain complex are paralleled by changes observed in components of the respiratory chain complex assessed by Western blotting (new figure 6).

**Comment 4.** The authors may need to compare the ratio of heart weight/tibia length, since body weight may be affected by tumor.

*Response:* We thank the reviewer for the comment. Unfortunately, animal carcasses are no longer available for us to make this determination.
