# Supplementary material for: Impaired cardiac performance, protein synthesis, and mitochondrial function in tumor-bearing mice
Source: PLoS One. 2019 Dec 18;14(12):e0226440. doi: 10.1371/journal.pone.0226440 (PMC6919625; doi:10.1371/journal.pone.0226440)
Supplement: Attachment — Submitted filename: Response to reviewers 11-11-2019.docx [file pone.0226440.s004.docx]

**Response to reviewers.**

**ALL CHANGES HAVE BEEN HIGHLIGHTED IN YELLOW IN THE REVISED MANUSCRIPT.**

**Reviewer #1:**

**Comments and responses:**

**Comment 1.** The authors have a adequately addressed my concerns. I appreciate that the author is added the statement of cardiac fibrosis, even though the author is did not observe any significant difference, I would suggest the author is added the figure evidence to the supplement.

*Response:* We have now added of figure (**Supplemental Figure 1**) showing the absence of fibrosis in cardiac tissue derived from tumor-implanted mice using the Masson Trichrome which is exceptionally sensitive for sensitive for fibrosis. As noted, we did not observe fibrosis in either the vehicle or tumor cell injected mice.

**Reviewer #2**

**Comment 1.** I want to thank the authors for addressing my concerns on technical issues. While I agree with the authors that this study provides certain novel insights about this type of tumor cells in triggering heart disease, the authors may consider discussing previous findings in a matter of comparing with current work

*Response:* Thank you for your comments. We have added sentences now in which we discuss our work in comparison to work previously published by others.
